# Sonographic Evaluation of Maternal Renal Echogenicity in Healthy Pregnant Women in the Niger Delta Region of Nigeria

**DOI:** 10.4314/ejhs.v33i3.10

**Published:** 2023-05

**Authors:** Peter Chibuzor Oriji, Enefia Kelvin Kiridi, Emily Gabriel Enefia Kiridi, Obiora Chibundu, Lukman Obagah, Johnpatrick Uchenna Ugwoegbu, Akaninyene Eseme Ubom, Panebi Yao Bosrotsi, Abednigo Ojanerohan Addah, Adedotun Daniel Adesina

**Affiliations:** 1 Department of Obstetrics and Gynaecology, Federal Medical Centre, Yenagoa, Nigeria; 2 Department of Radiology, Niger Delta University Teaching Hospital, Okolobiri, Nigeria; 3 Silhouette Radiodiagnostic Consultants, Yenagoa, Nigeria; 4 Department of Physiology, Niger Delta University, Wilberforce Island, Amassoma, Nigeria; 5 Department of Obstetrics and Gynaecology, Asokoro District Hospital, Nigeria; 6 Department of Obstetrics and Gynaecology, Nile University of Nigeria, Nigeria; 7 Department of Radiology, Federal Medical Centre, Owerri, Nigeria; 8 Department of Obstetrics, Gynaecology and Perinatology, Obafemi Awolowo University Teaching Hospitals Complex, Ile-Ife, Nigeria; 9 International Federation of Gynaecology and Obstetrics (FIGO) Committee on Childbirth and Postpartum Haemorrhage; 10 Department of Obstetrics and Gynaecology, Diete Koki Memorial Hospital, Yenagoa, Nigeria; 11 Department of Obstetrics and Gynaecology, Niger Delta University Teaching Hospital, Okolobiri, Nigeria; 12 Department of Medical Services, Nigerian Law School, Yenagoa Campus, Yenagoa, Nigeria; 13 Oasis Public Health Consulting Ltd, Yenagoa, Nigeria

**Keywords:** Maternal renal echogenicity, Age, Parity, Gestational age, BMI, Normal pregnancy

## Abstract

**Background:**

Increased renal echogenicity is a nonspecific ultrasound finding. It may be a normal variation or suggestive of various underlying conditions like renal amyloidosis, chronic kidney disease, sickle cell disease and HIV associated nephropathy (HIVAN).

**Objective:**

To study maternal renal echogenicity in normal pregnancy, and explore its relationship with maternal baseline characteristics in our subregion.

**Methods:**

This descriptive, cross-sectional study was conducted in the Obstetrics and Radiology Units of the two tertiary health facilities, one secondary facility and one radio-diagnostic facility, all in Bayelsa State, South-South Nigeria, between March-August 2022. The relationships between maternal renal echogenicity and age, parity and gestational age were explored using Chi-square test of proportion, while with an analysis of variance (ANOVA), the mean difference of age, weight and height between the grades of renal echogenicity was investigated. Kruskal Wallis test was deployed to examine parity in the grades of renal echogenicity. The level of significance was set at p<0.05.

**Results:**

The study participants that had Grade 0, 1 and 2 renal echogenicity were 160 (39.7%), 403 (58.3%) and 8 (2.0%), respectively. There were statistically significant relationships between maternal renal echogenicity and maternal age (χ^2^=36.94; p=0.001), parity (χ^2^=64.29; p=0.001), gestational age (χ^2^=16.03; p=0.003) and body mass index (BMI) (χ^2^ = 45.15; p – 0.001).

**Conclusion:**

Our study revealed a significant relationship between maternal renal echogenicity in normal pregnancy and maternal baseline characteristics (age, parity, gestational age and weight).

## Introduction

For evaluating human kidneys, ultrasound imaging is a crucial diagnostic technique. In this diagnostic technique, radiofrequency sound waves are sent into the body by an ultrasound transducer. These waves change as a result of their interaction with tissues and tissue surfaces, and they then reflect back to the transducer as echoes (1). In response, its piezoelectric crystals vibrate, converting the echoes into electrical signals (1). These signals are then processed using intricate algorithms to produce cross-sectional images of the body's underlying tissue layers. Maternal renal echogenicity in normal pregnancy has not been well-researched. Most of the reported studies on renal echogenicity in pregnancy are foetal (2–5). Increased renal echogenicity outside pregnancy is a non-specific observation. However, it can be indicative of normal variation or various underlying conditions like renal amyloidosis, chronic kidney disease (CKD), sickle cell disease and HIV-associated nephropathy (HIVAN) (1,6).

Both qualitative and quantitative methods can be used to assess the echogenicity of the renal cortex in comparison to the liver or spleen. However, qualitative methods are more frequently employed. Normal renal cortex is typically isoechoic (same brightness as the liver or spleen) or hypoechoic (less bright). CKD is frequently associated with increased cortical echogenicity, which has been linked to interstitial fibrosis, tubular atrophy, and glomerulosclerosis in histologic studies (7). However, inflammatory infiltrates and proteinaceous casts reflect sound waves, contributing to enhanced echogenicity in acute kidney injury (AKI) (e.g., acute glomerulonephritis, acute tubular necrosis) (7).

In addition to increased echogenicity, CKD (except diabetic nephropathy and infiltrative diseases) is typically linked with decreased kidney length and cortical thickness (7). The adult human kidneys typically measure 10 to 12 cm in length, though this varies depending on body size (7). The cortical thickness of the kidney is measured from the base of the medullary pyramid to the outer edge of the kidney. It typically ranges from 7 to 11 mm thick, being thicker near the poles (7). Since research on the subject matter is sparce, we sought to evaluate maternal renal echogenicity in normal pregnancy, and explore its relationship with maternal baseline characteristics (age, parity, gestational age and weight) in our sub-region

## Subjects and Methods

**Study design and setting**: This descriptive, cross-sectional study was conducted at the Obstetrics and Radiology Units of the Niger Delta University Teaching Hospital in Okolobiri, the Federal Medical Centre in Yenagoa, Silhouette Radiodiagnostic Consultants, and Diete Koki Memorial Hospital in Yenagoa, all in Bayelsa State, Nigeria, between March-August 2022. The first two study centres are tertiary health facilities that provide specialised gynaecological services to women in Bayelsa State, and serve as referral centres for other hospitals in Bayelsa State, and surrounding Rivers and Delta States, both in South-South Nigeria. The third study centre is a secondary health facility, while the fourth is the biggest radiodiagnostic facility in Bayelsa State, Nigeria.

**Sample size calculation**: The sample size for this study was calculated using the Fisher's formula (8). Four hundred and three consecutive eligible and consenting pregnant women were therefore enrolled for the study.

While women with normal singleton pregnancies, women with medical conditions like renal, hypertensive, diabetic, sickle cell disease or HIV in pregnancy were excluded.

Women who met the criteria for inclusion in the study were counseled, and a written informed consent obtained before being enrolled. The socioeconomic characteristics of the study participants were recorded, including age, marital status, and occupation. Gestational age was calculated from the last menstrual period, confirmed to correlate with the gestational age on early ultrasound. The heights and weights of the women were measured prior to the investigative modality. Urinalysis, serum electrolytes, urea and creatinine, and liver function tests were done for the women, and if these were normal, they were referred to the Radiology Units of the study centre for renal ultrasound scan.

**Procedure**: Protocols for placing patients and scanning them as outlined by Vanderwerff and Winter were used (9). Ultrasound scans were carried out transabdominally by four Consultant Radiologists (one for each study centre) with special interest in renal ultrasound scans. Before the research commenced, the four Consultant Radiologists convened to assess for interobserver variability and reliability, and agreed on the grading method for renal echogenicity to be used. Prior to data collection, ultrasound scans were performed for 20 participants to assess for interobserver variability and reliability. For every scan, a chaperone was present to ensure the woman's comfort and ease any possible anxiety she may experience during the procedure. With the woman in supine position on an examination couch, the abdomen and pelvis were exposed for the investigative modality, and adequate amount of ultrasound gel was applied to these exposed areas. The gel helped the transducer move more easily and removed air from the skin. We employed a 2012 Philips HD11 device with a 3.5 MHz curvilinear (convex) transducer (probe). With gain adjusted as necessary for acceptable image quality, the probe was moved back and forth on the skin and in orthogonal planes.

**Figure 1:**
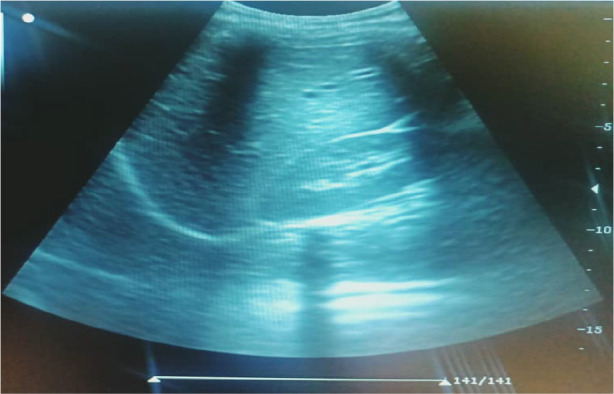
Grade 0 (from this study).

**Figure 2:**
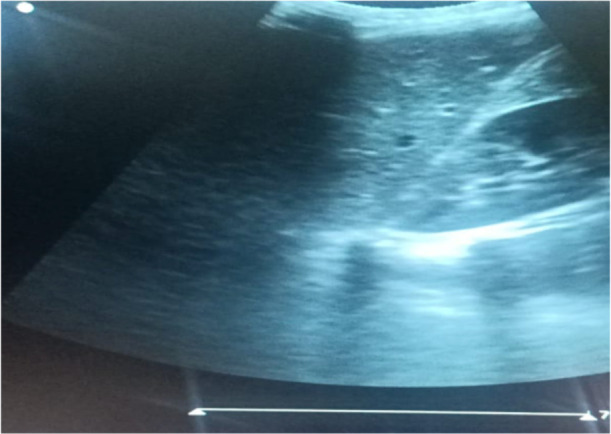
Grade I (from this study).

**Figure 3:**
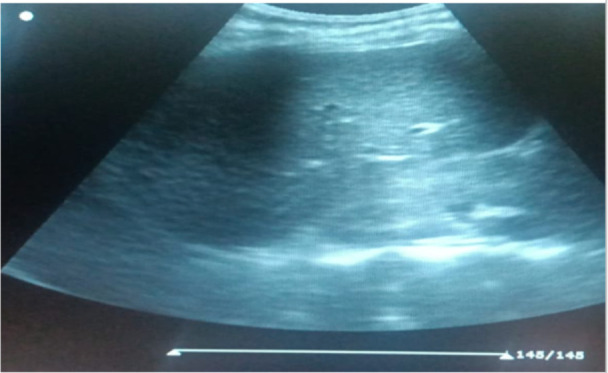
Grade II (from this study).

To image the right and left kidneys, respectively, patients were scanned in the right or left anterior oblique posture. Using Hricak *et al.'s* method(10), the kidney echogenicity was classified into Grade 0: kidney cortical echogenicity is slightly less than that of the liver (Figure 1). Grade 1: kidney cortical echogenicity is the same as that of the liver (Figure 2). Grade 2: kidney cortical echogenicity is moderately higher than that of the liver with moderate loss of corticomedullary distinction (Figure 3). Grade 3: kidney cortical echogenicity is much higher than that of the liver with complete loss of corticomedullary distinction.

**Data analysis**: Data was checked for correctness and completeness after every data collection exercise. Data were coded and entered into the Statistical Product and Service Solutions for Windows^®^ version 25, SPSS Inc.; Chicago, USA, which was used for both data cleaning and analysis. Univariate analysis included summarizing categorical variables like age-group, grouped parity, trimesters of pregnancy and renal echogenicity grading using frequencies and percentages and for continuous variables which included maternal age, weight and height, mean and standard deviation were used. Parity, an ordinal variable, was reported as median and range. The relationship between grade of renal echogenicity and age, parity and trimester of pregnancy was explored using Chi-square test of proportion, while with an analysis of variance (ANOVA) the mean difference of age, weight and height between the three grades of renal echogenicity was investigated. Kruskal Wallis test was deployed to examine parity in the three renal echogenicity grades. Interobserver and intraobserver variations were calculated with the use of the intraclass correlation coefficient (ICC) and documented. Level of statistical significance for this study was set at p < 0.05.

**Ethics**: The Research and Ethics Committee of the Federal Medical Centre, Yenagoa, Bayelsa State, Nigeria, gave ethical approval for this study (FMCY/REC/ECC/2022/656).

## Results

**Maternal baseline characteristics**: Four hundred and three pregnant women participated and completed the study. Of the 403 women, 144 (35.7%) were aged 25 – 29 years, while teenagers accounted for 7.9% of pregnant women. The mean age and median (IQR) parity of the study participants were respectively 28.7 ± 5.6 years and 1 (0-2). About two out of every five (41.9%) women were nulliparous, with most (186, 46.2%) of them in the second trimester of pregnancy. The mean weight and height of the women were 72.7 ± 15.0 kg and 1.6 ± 0.1 m, respectively (Table 1).

**Table 1:** Maternal baseline characteristics (n=403)

Characteristics	Frequency (%)
**Age (years)**	
<20	32 (7.9)
20 –24	80 (19.9)
25 – 29	144 (35.7)
30 – 34	82 (20.3)
35 – 39	65 (16.1)
**Mean age (years) ± SD**	28.7 ± 5.6
**Parity**	
Nullipara	169 (41.9
Primipara	98 (24.3)
Multipara	105 (26.1)
Grand-multipara	12 (3.0)
Median (IQR) parity	1.0 (0.0 – 2.0)
**Trimester of Pregnancy**	
First	40 (9.9)
Second	186 (46.2)
Third	177 (43.9)
**Maternal mean weight (kg) ±** **SD**	72.7 ± 15.0
**Maternal mean height (m) ±** **SD**	1.6 ± 0.1

**Figure 4:**
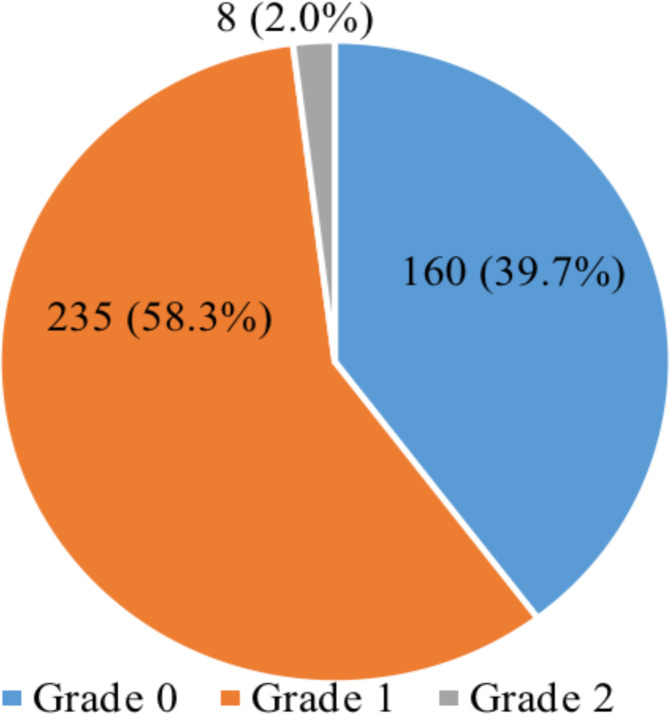
Grading of renal echogenicity among the participants.

**Grades of maternal renal echogenicity among the study participants:** More than one-half (403, 58.3%) of the study participants had Grade 1 renal echogenicity, 160 (39.7%) had Grade 0, while only 8 (2.0%) had Grade 2 echogenicity (Fig. 4).

***Maternal age, gestational age, parity, and anthropometric parameters across the different grades of renal Echogenicity:*** The mean age of women with Grade 0 renal echogenicity was 27.8 ± 5.2 years; those with Grade 1 echogenicity had a mean age of 29.3 ± 5.8 years, while the eight women with Grade 2 echogenicity had a mean age of 28.0 ± 2.3 years. There was a statistically significant difference (*f=3.40; p=0.034*) in the mean ages of the women across the different grades of renal echogenicity. There were also statistically significant differences in parity (*f =3.40; p=0.034*), gestational age (*f =5.05; p=0.007*) and maternal weight (*f=8.50; p=0.001*) across the three grades of renal echogenicity (Table 2). Interobserver and intraobserver variations were assessed with the use of the intraclass correlation coefficient (ICC), and the results were 0.99 (95% CI 0.38 – 0.99) and 0.98 (95% CI 0.40 – 0.99), respectively.

**Table 2:** Maternal age, gestational age, parity, and anthropometric parameters across the different grades of renal Echogenicity

Characteristics	Renal echogenicity			*f*-test (p-value)
		
	Grade 0 n=160 (%)	Grade 1 n=235 (%)	Grade 2 n=8 (%)	
**Age (years)**	27.8 ± 5.2	29.3 ± 5.8	28.0 ± 2.3	3.40 (0.034*)
**Parity**	1 (0 – 4)	1 (0 – 5)	1 (1 – 1)	6.02a (0.049*)
**Gestational age (weeks)**	25.8 ± 8.7	26.9 ± 7.3	18.5 ± 0.5	5.05 (0.007*)
**Weight (kg)**	74.9 ± 18.9	71.8 ± 11.4	54.0 ± 0.0	8.50 (0.001*)
**Height (m)**	1.64 ± 0.06	1.63 ± 0.05	1.66 ± 0.00	0.46 (0.633)

a Kruskal-Wallis test

**Relationship between maternal age, parity, trimester of pregnancy and renal Echogenicity**: Table 3 shows that there were statistically significant relationships between the grade of renal echogenicity and maternal age *(χ^2^=36.94; p=0.001)*, parity *(χ^2^=64.29; p=0.001),* trimester of pregnancy *(χ^2^=16.03; p=0.003)* and body mass index *(χ^2^ = 45.15; p -0.001)*. Fifty percent (16) of women aged 20-29 years had either Grade 1 or 2 renal echogenicity, which was present in 80.5% (66) of those between 30-34 years. The frequency of Grade 1 or 2 renal echogenicity increased from 52.7% (89) amongst nulliparous women to 87.1% (27) amongst grand-multiparous women. Renal echogenicity was lowest (Grade 0) in the first trimester of pregnancy (20, 50%) and highest (Grade 1 or 2) in the second trimester (126, 67.7%) (Table 3).

**Table 3:** Relationship between maternal age, parity, trimester of pregnancy and renal Echogenicity

Characteristics	Renal echogenicity			χ^2^ (p value)
		
	Grade 0	Grade 1	Grade 2	
	n=160 (%)	n=235 (%)	n=8 (%)	
**Age (years)**				
<20	16 (50.0)	16 (50.0)	0 (0.0)	36.94 (0.001*)
20 – 24	40 (50.0)	40 (50.0)	0 (0.0)	
25 – 29	64 (44.4)	72 (50.0)	8 (5.6)	
30 – 34	16 (19.5)	66 (80.5)	0 (0.0)	
35 – 39	26 (40.0)	39 (60.0)	0 (0.0)	
**Parity**				
Nullipara	80 (47.3)	89 (52.7)	0 (0.0)	64.29 (0.001*)
Primipara	24 (24.5)	74 (75.5)	8 (8.2)	
Multipara	56 (53.3)	49 (46.7)	0 (0.0)	
Grand multipara	4 (12.9)	27 (87.1)	0 (0.0)	
**Trimester of Pregnancy**				
First	20 (50.0)	20 (50.0)	0 (0.0)	16.03 (0.003*)
Second	60 (32.3)	118 (63.4)	8 (4.3)	
Third	80 (45.2)	97 (54.8)	0 (0.0)	
**Body mass index**				
Normal	48 (35.3)	80 (58.8)	8 (5.9)	45.15 (0.001*)
Overweight	72 (42.9)	96 (57.1)	0 (0.0)	
Mildly obese	24 (29.3)	58 (70.7)	0 (0.0)	
Moderately obese	16 (100.0)	0 (0.0)	0 (0.0)	

## Discussion

Echogenicity describes how bright or dark something looks in the gray-scale image; the brighter it appears, the more echogenic it is. In terms of the kidneys, echogenicity mainly relates to how bright or dark the right and left kidney parenchyma appear in comparison to the liver and the spleen, respectively (11). There is a paucity of published data on maternal renal echogenicity in normal pregnancy. What has been extensively studied is foetal renal echogenicity. Increased renal echogenicity in pregnancy may be a non-pathologic finding (12). Hydration in healthy persons without any underlying renal pathology may cause increased renal cortical echogenicity (12). All our study participants drank about four glasses of water, about an hour prior to ultrasound scan, in order to fill their urinary bladders, and create an optimal acoustic window. This possibly explains why up to 60% of the normal pregnant women in our study had increased renal echogenicity. Increased maternal renal echogenicity in pregnancy should therefore not be considered pathologic until physiologic causes are excluded.

Our study revealed that there was statistical significance between age and maternal renal echogenicity. This meant that the likelihood of a woman having echogenic kidneys in pregnancy increased with her age. The reason for this may not be unrelated to the fact that medical conditions that adversely impact the kidneys increase with age. The relationship between age and maternal renal echogenicity is only well-researched in the paediatric age group, where renal echogenicity reduces with increase in age until about the age of 15 years (13,14). Our observation contrasts those of Emamian *et al.*, who had previously observed in their study involving 665 adults, that right renal echogenicity reduced with increase in age, while that of the left remained almost the same with increase in age (15). The authors expressed concern about the standard to which the kidney is compared, which may not be consistent for both the right and the left kidneys. The standard (the liver) for comparison with the right kidney is different from the standard (the spleen) used for the left kidney (15). The echogenicity of the liver and that of the spleen are affected differently by age (15). Therefore, more research to provide a better and more reliable standard for comparison of renal echogenicity is recommended.

This study also observed that the relationship between parity and maternal renal echogenicity was statistically significant. The relationship between parity and maternal renal echogenicity, to the best of our knowledge, has not been well-researched. However, it has been observed that as parity increases, the frequency of renal and other metabolic conditions that adversely impact the kidneys rises (16,17). Pregnancy causes significant metabolic changes in the endocrine and immunological systems, which have a long-term impact on women's health (18).

There was also a statistically significant relationship between maternal obesity and maternal renal echogenicity in our study. This observation is in tandem with the finding of Faubel *et al.*, who observed that renal echogenicity increases with increase in obesity (11). The reason for this in not well understood. However, the deposition of fat on the kidneys in obese women may contribute to the increased echogenicity of the kidneys. Obesity is also a risk factor for kidney diseases (19).

The ICC was applied to reduce interobserver and intraobserver variability for the renal echogenicity measurements. The ICC evaluates the consistency of measurements of the same item made by various radiologists (20). ICC compares the variance of multiple data sets with the total of all measurements (21,22). The normal range is between 0 and 1, and a number greater than 0.8 indicates nearly perfect agreement (21,22). Our inter- and intra-observer variance values were 0.99 and 0.98, respectively, indicating a nearly perfect agreement. The variance of all measurements is taken into account by ICC in addition to the variations between the observers (20,23).

Our study is an important addition to the available body of sparse evidence on maternal renal echogenicity in pregnancy and its relationship to maternal baseline characteristics. Though a study limited to our region, it provides important data to guide the interpretation of renal echogenicity in pregnancy in our study population and provides a basis for larger scale, highly powered, multicentre/national studies that will be generalizable to our population.

There is paucity of data on maternal renal echogenicity in pregnancy. Whereas increased renal echogenicity may be associated with various pathologic renal conditions, it may also occur in the absence of any renal pathology, as evidenced in our study, where up to 60% of the women with normal pregnancies had increased renal echogenicity. Renal hyperechogenicity in pregnancy should therefore be interpreted with caution, and physiologic causes, including hydration, excluded. Our study revealed statistically significant relationships between maternal renal echogenicity and maternal age, parity*,* gestational age, and body mass index. Maternal baseline characteristics should therefore be considered when interpreting renal hyperechogenicity in pregnancy.
